# Supplementary material for: Serrate RNA Effector Molecule (SRRT) Is Associated with Prostate Cancer Progression and Is a Predictor of Poor Prognosis in Lethal Prostate Cancer
Source: Cancers (Basel). 2023 May 22;15(10):2867. doi: 10.3390/cancers15102867 (PMC10216025; doi:10.3390/cancers15102867)
Supplement: Supplementary file 1 [file cancers-15-02867-s001.zip › cancers-2309004-supplementary.pdf]

Supplemenatry Data

Table S1. Study group classification and OS and CSS variables.

Group	N(%)	OS Event (all-cause mortality)	Median Followup	Median Overall Survival	p-value	Cause-specific survival events	Median CSS	p-value
Incidental	182 (38.5)	83	59.10 (0.79 - 154.97)	92.78 (71.51-114.05)	<0.0001	5	NR	<0.0001
Advanced	178 (37.6)	139	35.99 (0.30-147.55)	38.60 (31.04-46.16)		80	63.97 (53.04-74.89)	
Castrate Resistant	113 (23.9)	104	16.00 (1.54-109.24)	16.23 (13.10-19.36)		81	18.53 (13.76-23.30)	
Overall	473	326	39.06 (0.30-154.97)	42.48 (37.73- 7.22)		166	NR	

NR No reported death.
